# Advanced Bioactive Polymers and Materials for Nerve Repair: Strategies and Mechanistic Insights

**DOI:** 10.3390/jfb16070255

**Published:** 2025-07-09

**Authors:** Nidhi Puranik, Shraddha Tiwari, Meenakshi Kumari, Shiv Kumar Yadav, Thakur Dhakal, Minseok Song

**Affiliations:** 1 Department of Life Sciences, Yeungnam University, Gyeongsan 38541, Gyeongbuk, Republic of Korea; nidhipuranik30@yu.ac.kr (N.P.); shraddha_tiwari@yu.ac.kr (S.T.); 2 Department of Botany, Career Point University, Kota 324005, Rajasthan, India; meenakshi.kumari@cpur.edu.in; 3 Department of Botany, Government Lal Bahadur Shastri PG College, Sironj, Vidisha 464228, Madhya Pradesh, India; shivkumar.kumar741@gmail.com

**Keywords:** bioactive materials, nerve repair, neuroprosthetics, immune response

## Abstract

Bioactive materials have recently shown potential in nerve repair and regeneration by promoting the growth of new cells, tissue repair, and restoring nerve function. These natural, synthetic, and hybrid materials offer a biomimetic structure, enhance cell attachment, and release bioactive molecules that promote the axonal extension of severed nerves. Scaffold-based preclinical studies have shown promising results on enhancing nerve repair; however, they are limited by the immune response and fabrication, scalability, and cost. Nevertheless, advances in manufacturing, including 3D bioprinting, and other strategies, such as gene editing by CRISPR, will overcome these shortcomings. The opportunity for the development of individualized approaches and specific treatment plans for each patient will also increase the effectiveness of bioactive materials for the treatment of nerve injuries. Combining bioactive materials with the neural interface can develop new reliable therapeutic solutions, particularly for neuroprosthetics. Finally, it is essential to stress a multidisciplinary focus, and future studies are needed to enhance the potential of bioactive materials for patients with nerve injuries and the field of regenerative medicine.

## 1. Introduction

Peripheral nerve injuries can range from minor injuries to the complete severing of nerves [1]. These injuries may occur from trauma, surgical complications, or illness and may be characterized by motor, sensory dysfunction, or pain [2]. Peripheral nerves exhibit a capability to regenerate, but this is a slow process and the regeneration is seldom complete, particularly if the distance between severance extremities is large [3,4]. Stem cell treatment becomes even more challenging when applied to central nervous system (CNS) injuries because adult CNS neurons do not have regenerative capacity. There are also myelin inhibitory factors present in the CNS environment [5]. The cost implications of nerve injuries are enormous. Patients require more care and focus on their social and economic rehabilitation. Sometimes surgery is required, which may not always work as expected. These issues underscore the importance of developing fresh approaches to improve nerve repair as well as regeneration capacity [6]. Despite the availability of nerve grafts and end-to-end anastomosis, current clinical options may not be sufficient in reestablishing full functionality [7,8,9]. Limitations to autologous nerve grafts, which are the gold standard of nerve reconstruction, include donor site morbidity, limited supply, and a fixation on the structure or size of the nerve. In addition, nerve repair and regeneration are complex, with multiple cells and signaling pathways that regulate the directions of axon outgrowth, reconnection with target organs and tissue, and the re-establishment of functional synapses [10,11]. One of the main reasons for regeneration failure is the formation of a dense connective tissue scar, the absence of stimulating signals for tissue regeneration, and the weakness of support from the extracellular matrix. These challenges require the identification of new therapies that enhance outcomes for patients with nerve injuries [12]. Bioactive materials have now been identified as a potential solution for these complications. These materials exert a benign response to biological tissue and have unique properties that are beneficial for nerve repair. Compared with the passive approach of using materials that do not affect cell function, bioactive agents can directly stimulate the proliferation, migration, and differentiation of cells that are involved in nerve regeneration [13]. These materials can replicate the natural extracellular matrix to promote axonal growth and direct regenerating nerves to the target tissues as they offer structural support. In addition, they enable signaling molecules, growth factors, and drugs on the surface of bioactive materials and promote the remodeling and healing potential of the tissue [14]. The significance of bioactive materials transcends functionality. They point to more complex, cross-disciplinary, regenerative medicine strategies [15,16]. Advances in material science, biotechnology, and nanotechnology have facilitated the development of materials that are ideal for particular tasks in nerve repair [17]. These include hydrogels [18], scaffolds [19], and nanoparticles that may be engineered to release bioactive factors or provide electrical stimuli typically observed in tissues. Furthermore, bioactive materials provide an opportunity to harness highly developed engineering and electronics tools, such as 3D bioprinting and neural devices, thus creating a brand-new paradigm for individualized treatment [20]. Bioactive materials continue to be unique to the field of regenerative medicine, particularly given the many drawbacks of current nerve repair methodologies [15]. Their ability to fill the knowledge gap raises hope for improved outcomes, fewer complications, and enhanced quality of life for patients with nerve injuries [21]. Advances in the development of these materials for nerve repair and regeneration have raised significant interest among researchers and clinicians [22].

## 2. Nerve Repair and Regeneration

**Figure 1:**
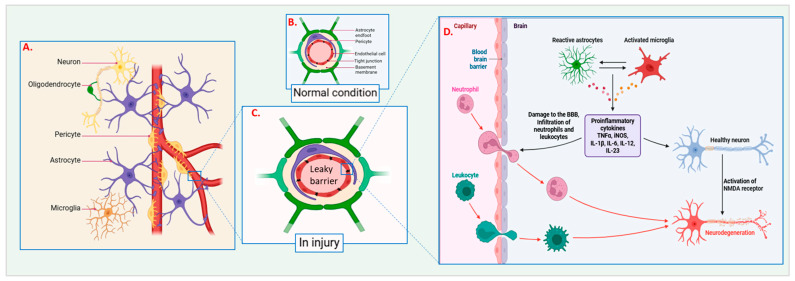
Cellular and molecular responses in the CNS under normal and injured conditions. (**A**) Major CNS Cell Types: Diagram illustrating key cellular components of the CNS, including neurons, oligodendrocytes, pericytes, astrocytes, and microglia, in proximity to the brain vasculature. (**B**) Normal Condition: Under physiological conditions, the BBB remains intact due to the structural and functional support of astrocytic endfeet and pericytes surrounding endothelial cells, maintaining tight junctions and selective permeability. (**C**) Injury-Induced BBB Disruption: Following CNS injury, the integrity of the BBB is compromised, leading to increased permeability and the formation of a leaky barrier that permits the infiltration of harmful substances and immune cells. (**D**) Pathological Cascade Following BBB Disruption: Injury-induced BBB damage results in the transmigration of neutrophils and other leukocytes into the brain parenchyma. Reactive astrocytes and activated microglia release pro-inflammatory cytokines, including TNF-α, iNOS, IL-1β, IL-6, IL-12, and IL-23. This neuroinflammatory environment contributes to excitotoxicity through NMDA receptor overactivation and leads to neurodegeneration, contrasting with healthy neuronal conditions. Reprinted from Ref. [33].

Surgery for nerve repair and regeneration is a delicate process, in which the anatomy and physiology of a damaged nerve are reconstructed [23]. The processes by which these events occur are distinct depending on whether the peripheral nervous system (PNS) or the CNS is involved; however, the PNS has a greater capability of regeneration compared with the CNS [24]. For the PNS, Schwann cells are central in the regenerative response to an injured nerve. They secrete neurotrophic factors and also form bands of Büngner, which are channels formed by Schwann cells and macrophages, and promote regenerating axons to grow toward the target tissue (Figure 1). Moreover, macrophages clear the myelin debris, which is essential to regenerating the myelin sheath. Of these two systems, the CNS has a relatively poor reparative capacity because of inherent chemical barriers, a dearth of powerful supporting cells, and the development of glial scars that hinder axonal growth [21]. Understanding these mechanisms is important given the need to create strategies that may improve nerve repair and regeneration in both systems. Many variables affect the ability of injured nerves to regenerate, including the severity of the damage to the surrounding environment at the injury site [25,26,27]. Thus, the extent of nerve injuries, such as axonal, transected, or crushed ones, predicts the extent of regeneration. In addition, other factors, including age, general health, and factors such as diabetes or infection, can impair the body’s ability to repair nerves. The overall local environment at the site of the injury, such as the level of growth-promoting and -inhibiting factors, affects the extent of recovery [28]. The molecules that promote regeneration include nerve growth factor (NGF) and brain-derived neurotrophic factor (BDNF) [29,30], whereas the inhibitory molecules are myelin-associated glycoproteins [31]. The immune response, while important for clearing debris and removing infected tissues, should be regulated and not allowed to hinder the process of regeneration. To support nerve regeneration, biological and mechanical parameters have to be fulfilled. This is especially true for the extracellular matrix, which needs to be established [23]. This means providing signals at the biochemical level that promote cell attachment, growth, and specialization to the surrounding structures. Remyelination is required to establish a framework for axonal growth, and Schwann cells must also be activated [23]. In addition, neurotrophic factors must be delivered to the site of injury to promote regeneration over the long term. From a mechanical perspective, the issue of the alignment of regenerating axons is important [24]. Malposition can result in the formation of aberrant neural linkages, producing complications, such as neuroma formation or even a detrimental influence on functional improvement. Tissue tension at the injury site, biomechanical support, and the use of appropriate grafts or conduits that span the distance of the injury are also necessary [24]. Biological and mechanical approaches contribute to efficient nerve repair methodologies. It is often difficult to incorporate these requirements into a unifying therapy for nerve repair. For example, nerve grafts have mechanical properties that facilitate axonal regeneration in addition to acting as a conduit for the growing tips of the nerve fibers; however, other issues, such as biological compatibility and permissiveness for cell growth, must be considered [32]. Biomaterials and tissue engineering actively seek to fill this gap by designing and creating lateral and interpositional scaffolds and conduits that offer both mechanical support and bioactivity. They are hydrogels designed to mimic the properties of actual tissue, being soft and elastic, and polymers that can be functionalized for additional interactions with cells [32]. Furthermore, electrical stimulation is capable of fostering axonal process reorientation, which is another factor that complicates the criteria for effective regeneration. Thus, an understanding of how biological and mechanical factors promote nerve repair is fundamental when striving to improve patient outcomes following nerve injury surgery [32]. Identifying the factors that affect these recovery goals and how they may be enhanced provides a foundation for a new generation of therapies. Combining these applied sciences with novel technologies offers the possibility of refining regenerative potential to avoid the false positives and negatives that remain inherent in translating regenerative medicine to patients with paralyzing nerve injuries.

## 3. Bioactive Materials: Definitions and Properties

**Figure 2:**
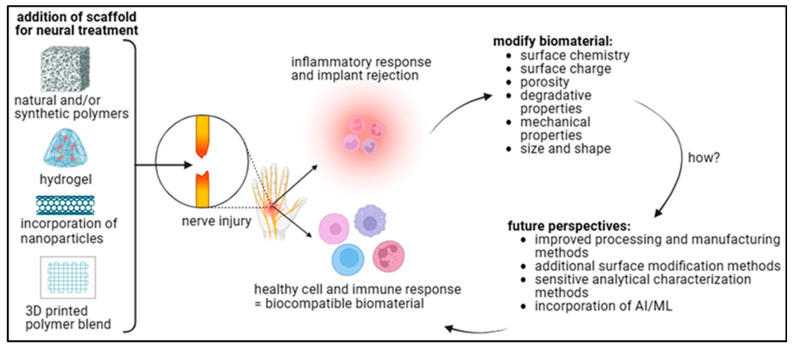
Strategies and considerations for scaffold-based biomaterial design in neural tissue repair. The schematic illustrates the use of various scaffold types—including natural and/or synthetic polymers, hydrogels, nanoparticles, and 3D-printed polymer blends—for neural injury treatment. Following nerve injury, scaffold implantation may trigger either a detrimental inflammatory response and implant rejection or a favorable interaction resulting in a biocompatible response from healthy cells and immune components. To enhance scaffold compatibility and therapeutic efficacy, key biomaterial properties can be modified, including surface chemistry, surface charge, porosity, degradability, mechanical properties, and size/shape. Future directions involve improving processing and manufacturing techniques, developing advanced surface modification strategies, employing sensitive analytical methods, and leveraging artificial intelligence/machine learning (AI/ML) to optimize scaffold design and performance. Reprinted from Ref. [40].

Bioactive materials are synchronized to interact directly with living tissues to induce definitive cell reactions and stimulate healing or tissue formation [34]. Although the major functions of conventional materials are to passively support the load and provide a scaffold, bioactive materials interact with cells, promote cell responses, and act as a vehicle to deliver molecules, such as growth factors or drugs (Figure 2). They may be categorized as natural, synthetic, or composite materials [35]. Most are obtained from natural sources, such as collagen, chitosan, and alginate, which show good compatibility and resemble the extracellular matrix. PGA and PCL represent synthetic polymers that are manufactured to have controllable mechanical and degradation characteristics [36]. Naturally sourced composite materials for load-bearing applications mimic some natural characteristics to achieve superior outcomes in nerve regeneration. For nerve regeneration, some properties of bioactive materials are more important [36]. Biocompatibility is a critical attribute, in which the material must be highly compatible with the immune response and accepted within the human body. Another important characteristic is biodegradability, in which the material turns into a harmless substance during the healing process [37]. This ensures that the tumor does not have to be surgically removed and helps to minimize the chances of encountering serious long-term implications [38]. Viability, or the capacity of a material to engage in chemical and/or physical interactions with neighboring cells and/or tissues to stimulate the appropriate cell processes, such as proliferation, differentiation, and migration, is important for these bioactive materials [39].

Mechanical properties, such as elasticity and tensile strength, should be carefully adjusted so materials can withstand the pressure of the surrounding nerve tissues while preserving the capacity to support regeneration [41]. Conductive materials are not essential but are highly recommended for nerve reconstruction because such surfaces provide pathways for electrical signaling and help to orient axons for growth [41]. An overview of the bioactive materials used in regenerative medicine is also important to demonstrate the richness of the field along with further perspectives. For natural materials, collagen is popular because of its affinity for the ECM and its stimulating effect on cell attachment and proliferation. Chitosan, which is a polymer of glucosamine derived from chitin, exhibits good biocompatibility and antibacterial properties; thus, it is preferred for nerve conduits [42]. Alginate is derived from algae and allows the formation of hydrogels that closely resemble the soft hydrated environment of nerves. Aliphatic polyesters, such as PLA, PGA, and PCL, are preferred because they have adjustable mechanical properties and predictable degradation profiles [2]. These polymers are usually combined with natural compounds to improve performance. Collagen–PCL composites take advantage of the characteristics of both phases by balancing bioactivity where mechanical stability is required [43]. Progress in nanotechnology has resulted in the development of nanoparticles or nanofibers that carry bioactive molecules or stimuli to regulate nerve repair. Because of the multifunctional nature of bioactive materials, they play an important role in regenerative medicine, particularly in addressing such challenging tissues as nerves [44]. The ability to incorporate biological, chemical, and biomechanical characteristics makes composites unique because they present singular opportunities to confront the complex issues of nerve regeneration [44]. Thus, these materials facilitate the creation of novel concepts for studying disease and treatments, which cannot be achieved with conventional tools and methods. Research and development will improve the prospects of regenerative medicines for patients with nerve injury.

## 4. Bioactive Materials: Nerve Repair

Nerve conduits have received attention in the area of regeneration because of the fabrication of bioactive materials [45]. From these, natural bioactive polymers, such as collagen, chitosan, and silk fibroin, have garnered interest because of their inherent biocompatible nature and the capacity to mimic the ECM. Of the various proteins that comprise the extracellular matrix, collagen supports cell adhesion, migration, and proliferation, which makes it ubiquitous among the scaffolds and conduits required for the nerve repair process [45]. Its structure offers direction to the growth of axons and supports Schwann cell function. Similarly, chitosan, a nontoxic cationic natural polysaccharide polymerized from chitin, shows excellent cytocompatibility and intrinsic antibacterial activity [46]. Expanded P2 XP has the characteristic of forming hydrogels and porous scaffolds, making it suitable for use where a soft, hydrated matrix is needed. Silk fibroin is derived from silkworm cocoons. It is biocompatible, has remarkable mechanical strength, and supports nerve regeneration, while encouraging cellular adhesion. However, synthetic bioactive materials, such as polyglycolic acid (PGA) and polycaprolactone (PCL), have salutary features, including controllable mechanical strength, degradation, and porosity. PGA is a biodegradable polymer that is widely used in tissue engineering because of its ability to provide a short-term scaffold matrix that disappears as new tissue forms [47]. It is used in nerve conduits to fill the residual spaces following nerve injury and provides mechanical strength during regeneration. Another humanmade polymer is PCL. Because of its slow degradation and flexible nature in the body, it is more suited to long-term applications in nerve regeneration [48]. Filled materials may also be chemically designed to contain bioactive molecules, such as growth factors or drugs, which increase their efficiency [49]. They may be designed to yield reproducible characteristics and properties that overcome some of the disadvantages of natural materials, such as poor reproducibility between batches [49]. The replacement of a portion of the synthetic part with the natural part in the construction of hybrid materials offers a possible solution for addressing challenges associated with the characteristics of these two material groups. Hybrids contain the bioactivity and biocompatibility of natural materials with the mechanical stability and adjustable characteristics of synthetic materials and represent an optimal repair solution for nerve injuries. For example, collagen–PCL composites combine the ability to support collagen cells with the structural support provided by PCL [49]. Similarly, the combination of chitosan–PGA improves both the bioactivity and mechanical properties of the conduits to benefit severed nerves. Some of these materials erode and can be modified to have preprogrammed degradation profiles for the steady release of bioactive agents over the entire regeneration phase [50]. Bioactive materials can be used for nerve repair and reconstruction, not only to provide mechanical support. They exhibit additional characteristics, such as electrical conductivity, to provide electrical signals necessary for nervous activity [50]. For example, conductive polymers such as polypyrrole have been introduced into hybrid scaffolds to induce axonal regrowth via electrical signals. Although these materials are biocompatible and biodegradable, they can be engineered to deliver growth factors and drugs to the injured area. The inclusion of natural, synthetic, and hybrid materials in the current designs provides new therapies that could restore the original functions of nerves. In the cases of tissue engineering, scaffold materials that are biodegradable are desirable and have made bioactive materials indispensable for regenerative medicine [51]. These proteins may come from natural sources. They can also be manufactured in the lab, and the combination of both may overcome the complex problems associated with nerve repair. Their continued development will alter the management of and recovery from nerve injuries, thus improving patients’ quality of life.

## 5. Natural Bioactive Material

### 5.1. Collagen

Collagen is the most abundant bioactive material derived from natural sources in regenerative medicine. It is known as a major structural protein of the ECM in the connective tissue and occupies a central position in determining the structural and microenvironment of biological tissues [52]. It is biocompatible in its inherent support of many cellular functions, including adhesion, migration, and proliferation, and is well-suited for tissue repair and regeneration. Hydrogels, sponges, and fibers represent categories of collagen-based materials that can be engineered and used for nerve repair [52]. Another advantage of collagen is that it is biomimetic in application. It supports a natural structure that mimics the native ECM and exhibits topographical and molecular signals that promote cell adhesion and proliferation. During nerve repair, collagen scaffolds or conduits can fill in spaces in severed nerves and direct axon regeneration toward target tissues without the formation of scar tissue that could otherwise hinder rehabilitation [53]. This establishes proper reorientation and functional regeneration, which is key in the rewiring of nerve fibers. Collagen is also hydrolyzable, which is an added advantage. It decomposes into nontoxic compounds that can be readily metabolized in the body during the natural healing process. The degradation rate can be regulated by chemically bonding collagen molecules together or combining them with other materials so the nerve scaffold can offer suitable support throughout the healing process [52,54]. Moreover, collagen is fully modifiable through the incorporation of bioactive agents, including growth factors, peptides, or drugs, thus broadening its therapeutic applications. Collagen is the best bioactive candidate for nerve repair in regenerative medicine because of its structural and functional similarity to the ECM [54]. As a primary structural protein in connective tissues, collagen offers a favorable locale that enhances cell adhesion, migration, proliferation, and differentiation. These characteristics mimic the conditions required for tissue healing and regeneration; therefore, they are an important factor in test designs for nerve regeneration [52]. Collagen-based substances include sponges and fibers that can be used for bridging nerve cuffs and axonal growth promotion. The mechanism by which collagen promotes nerve repair mimics that of the substratum. Thus, collagen reconstructs the morphological characteristics of the ECM and provides an interface for interactions with cells implicated in the regeneration process [14]. When the collagen scaffolds are applied to the site of a nerve injury, they offer a structure in which the regenerating axons grow toward the target tissue rather than forming neuromas [55,56]. The outer surface contains integrin-binding sites, which enable cell surface receptors to promote cellular contact with the ECM. These integrin-dependent interactions involve cell adhesion and the regulation of other pathways that are important to cell survival, migration, and differentiation. Another function of the glial fibrillary acidic protein is the regulation of the immediate stroma to promote nerve repair. It binds and externalizes growth factors, such as NGF and BDNF, while protecting and presenting them in a functional conformation [57]. Such interactions increase the local concentration of neurotrophic factors that activate Schwann cells and support axonal sprouting. Schwann cells play an important role in the nerve regeneration process because they form bands of Büngner to orient the regenerating axons and secrete growth factors required for the repair process [57]. In addition to its biochemical properties, collagen is biodegradable, resulting in harmless peptides and amino acids. This type of degradation is inherent, corresponds to the recovery timeline, and offers additional support while the regenerating nerve tissue stiffens [58]. The degradation rate can be adjusted by cross-linking the polymer or by compounding it with other polymers to match the requirements for regeneration [58]. In addition, natural or recombinant collagen scaffolds can be further surface-modified with extra bioactive molecules or drugs to overcome some specific issues in the regeneration of nerves, including scarring or inflammation [59]. For some specific uses, however, the mechanical characteristics of collagen, as a soft tissue engineering scaffold material, may need to be reinforced [59]. Its poor resistance to enzymatic degradation provides additional enzymatic activity; however, the issues of biodegradability and mechanical properties remain a limitation to the use of collagen, primarily because of the inability to access mechanically tangible substances post-treatment [60]. However, by making changes to the cross-linking mechanism and hybrid material formulation, collagen becomes stronger and more versatile for nerve repair.

### 5.2. Chitosan

Chitosan is a nontoxic, biodegradable, and biocompatible bioactive natural polysaccharide derived from chitin. It has been used as a viable bioactive material for tissue engineering, particularly for nerve regeneration [61]. It is biocompatible, biodegradable, and has certain properties that are important for tissue engineering. Morphologically, chitosan is similar to glycosaminoglycans, which are major components of the ECM, and can interact with cells and tissues [61]. These forms include hydrogels, scaffolds, membranes, and fibers, which create infinite possibilities for chitosan use. Moreover, because of its inherent antimicrobial properties and wound healing ability, it is suitable for nerve regeneration [62]. This biological effect of chitosan in nerve injury is based on the development of a conducive milieu that encourages cell attachment, migration, and proliferation for tissue repair and regeneration [62]. Chitosan scaffolds or conduits also offer a framework upon which a regenerating nerve is formed, which span gaps in severed nerves and become aligned along axonal processes. Chitosan is a cationic biopolymer manufactured from N-deacety chitin. The positively charged amino groups exhibit strong affinity towards negatively charged cell membranes and biomolecules, while promoting cell adhesion and activation [63]. This interaction also contributes to cross-linking and entrapment to promote the immobilization and stabilization of growth factors and allow for their steady release at the injury site. Thus, chitosan stimulates Schwann cells, which are needed for nerve regeneration [63]. Schwann cells are also involved in the assembly of bands of Büngner that are essential for directing axonal regrowth and for the synthesis of neurotrophic factors, such as NGF. We found that chitosan promotes the proliferation and migration of Schwann cells, thus contributing to nerve regeneration [64]. Chitosan may be modified or mixed with other molecules to increase its biological activity. For example, it can be chemically altered to include peptides, growth factors, or signaling molecules that further promote regeneration [65,66]. Another important property of chitosan is its biodegradability, which results in the formation of nonhazardous substances, including glucosamine, which are readily used by the body. To control the degradation rate, the degree of deacetylation may be altered or it may be blended with other materials [65]. This chrono-degradable property is necessary to provide mechanical support during the early periods of nerve regeneration and biodegradation during subsequent stages of tissue healing [65]. Another advantage of chitosan is its versatility in the formation of hydrogels and porous scaffolds for drugs or active biomolecules and its ability to limit inflammation or infection at the site of injury [67]. Chitosan also exerts antioxidant activity and decreases oxidative stress, which occurs during nerve injury. Scavenging free radicals protects cells and tissues from oxidative damage, thus improving conditions for nerve regeneration [67]. Moreover, chitosan has antimicrobial properties, which reduce the risk of infection and promote the healing process [67]. Despite these benefits, chitosan has some limitations. For example, it has low mechanical characteristics and is insoluble in neutral/basic media. These limitations may be overcome by altering the chemistry of the hydrogel, copolymerizing it with other polymers, or designing composite/hybrid systems [68]. Recent improvements in nanotechnology have introduced nanoparticles or nanofibers into chitosan-based systems to improve mechanical properties and functionality.

### 5.3. Silk Fibroin

Silk fibroin, a naturally occurring protein obtained from the silkworm cocoons of *Bombyx mori*, has emerged as a candidate material for regenerative medicine, particularly for nerve tissue regeneration [69]. It exhibits superior mechanical properties, biocompatibility, and biodegradability, which are important for tissue engineering applications. It may be processed into different forms, such as scaffolds, films, hydrogels, and fibers, depending on the regeneration requirements. Furthermore, silk fibroin exhibits very low immunogenicity, making it a safe implantable material [70]. The benefit of applying silk fibroin for nerve injury healing is based on its capacity to establish a scaffold for restoring the nerve, which can imitate ECM function. For nerve regeneration applications, silk fibroin scaffolds provide regenerating axons with structural predirection for preferential growth [71]. The surface chemistry of the material promotes cell adhesion, which enables neural cells and Schwann cells to attach and grow in abundance. Schwann cells play a role in regeneration as they release molecules that promote axonal elongation together with neurotrophins, such as NGF and BDNF [2]. Schwann cells are stimulated by silk fibroin to produce a regenerative milieu at the site of injury. The flexibility and range of hardness of silk fibroin render it particularly suitable for nerve regeneration [2]. Its tensile strength and elasticity can be tailored to match the requirements of the target tissue. For example, the mechanical stability of silk fibroin scaffolds ensures the maintenance of structural integrity during the critical phases of nerve regeneration, even under physical stress, while its flexibility allows it to adapt to the dynamic environment of nerve tissues [72]. This combination of properties ensures that the material provides the appropriate support for regenerating nerves. Another beneficial feature of silk fibroin is its biodegradability. It is degraded into nontoxic byproducts, such as amino acids, which can be metabolized or reused by the body [72]. The degradation rate of silk fibroin can be altered by cross-linking or blending it with other materials. This ensures that the scaffold provides sustained support during tissue regeneration. This controlled degradation prevents premature breakdown while avoiding long-term foreign body reactions. Silk fibroin can also be functionalized to enhance its activity [73]. It may be combined with growth factors, peptides, or drugs to further promote nerve repair. For example, scaffolds can be loaded with NGF or BDNF to enhance axonal sprouting and neuronal survival. The ability of fibroin to deliver bioactive molecules in a controlled manner ensures that the injury site remains enriched with essential factors throughout the healing process. In addition to its role as a physical scaffold, silk fibroin exhibits inherent biocompatibility and supports the formation of vascularized networks, which are required for providing oxygen and nutrients to regenerating tissues. Its unique structure allows for the incorporation of nanoscale features that mimic the natural ECM, further enhancing its ability to support cell growth and differentiation [74].

Silk fibroin is processed into multifunctional formats, such as hydrogels, bioelectronic interfaces, films, nanoparticles, nanofiber bandages, membranes, and sponges. They exhibit excellent biocompatibility, mechanical strength, and biodegradability, enabling their use in tissue engineering and regenerative medicine. The figure highlights targeted therapeutic applications in bone repair, ocular regeneration, skin healing, tendon repair, and nerve regeneration, which highlight the versatility and adaptability of silk fibroin-based systems.

## 6. Synthetic Bioactive Materials

### 6.1. Polyglycolic Acid

PGA is a biodegradable, synthetic polymer that has gained significant attention as a bioactive material for nerve repair and tissue engineering applications. It is an aliphatic polyester, and its properties, including biodegradability, biocompatibility, and tunable mechanical characteristics, make it suitable for use in regenerative medicine [75]. PGA can be processed into various forms, such as fibers, meshes, and scaffolds, which enable its application in nerve repair, wound healing, and drug delivery systems. Its degradation into nontoxic byproducts further enhances its suitability for clinical use, as it minimizes the risk of long-term foreign body reactions [76]. Its mechanism of action in nerve repair is based on its role as a scaffold material that supports cell growth, axonal regeneration, and tissue remodeling [77]. When used for nerve regeneration, PGA scaffolds provide structural support to guide the regeneration of axons across nerve gaps, enabling the proper alignment of regenerating fibers. The porous structure of PGA promotes the infiltration of cells, such as Schwann cells and fibroblasts, which are essential for tissue repair [78]. PGA scaffolds support cell adhesion and migration, which are necessary for initiating and sustaining nerve regeneration [79]. Schwann cells have a particularly important role in peripheral nerve regeneration by secreting neurotrophic factors, such as NGF and BDNF, which support axonal growth and survival [80]. Biodegradability is a key feature of PGA that makes it suitable for nerve repair applications. It undergoes hydrolytic degradation into glycolic acid, which is a naturally occurring metabolite that can be safely absorbed or excreted by the body. The degradation rate of PGA in polymer form can be adjusted by altering its molecular weight or by blending it with other materials [81]. This controlled degradation ensures that PGA scaffolds provide structural support during the critical stages of nerve regeneration and degrade as the tissue heals, which reduces the need for surgical removal. The mechanical properties of PGA are an important aspect of its performance during nerve repair. PGA is a relatively stiff polymer, which is advantageous for certain applications, in which mechanical stability is required; however, its rigidity may be a limitation in softer tissue environments, such as nerves. To address this limitation, PGA is often blended with other materials, such as poly (lactic acid) (PLA) or polycaprolactone (PCL), to improve its flexibility and mechanical performance. Hybrid materials combining PGA with natural biomaterials, such as collagen or chitosan, enhance the mechanical properties and biological activity of the scaffold. Its ability to support the controlled release of bioactive molecules further enhances its regenerative potential. Growth factors, such as NGF or vascular endothelial growth factor (VEGF), can be encapsulated within PGA scaffolds and released in a controlled manner to promote nerve regeneration and tissue vascularization. Erythropoietin-loaded PLGA-PEG was used as a local treatment to promote the functional recovery and neurovascular regeneration following peripheral nerve injury, which provides promising preclinical evidence for nerve regeneration [82]. In addition, PGA can be functionalized with peptides or other bioactive agents to stimulate specific cellular responses and improve the overall outcome of nerve repair [81].

### 6.2. Polycaprolactone (PCL)

PCL is a synthetic, biodegradable polymer recognized for its potential in nerve repair and tissue engineering applications. Its exceptional biocompatibility, slow degradation rate, and mechanical versatility make it a useful bioactive material for regenerative medicine [83]. PCL may be fabricated into various forms, including scaffolds, nanofibers, and hydrogels, making it useful for a variety of clinical applications. Its hydrophobic nature and ability to blend with other materials further enhance its utility, thus enabling the development of hybrid scaffolds with improved biological and mechanical properties. The effect of PCL on nerve repair is primarily based on its role as a supportive scaffold that facilitates axonal regeneration, cell adhesion, and tissue remodeling. PCL scaffolds provide a three-dimensional structure that bridges gaps in damaged nerves and guides regenerating axons toward their target sites [84,85]. The smooth and porous architecture of PCL scaffolds promotes the infiltration of Schwann cells and fibroblasts, which are critical for the repair process [86]. Schwann cells play an important role in peripheral nerve regeneration by forming bands of Büngner, secreting neurotrophic factors, such as NGF and BDNF, and myelinating regenerating axons. The slow biodegradation of PCL is one of its main advantages in nerve repair. Unlike other biodegradable polymers, PCL degrades over a prolonged period through hydrolytic cleavage of its ester bonds to produce nontoxic byproducts. This extended degradation profile ensures that the scaffold provides mechanical support for a longer duration, which is particularly beneficial to nerve regeneration, which can take several months [84]. The gradual degradation of PCL minimizes the risk of inflammatory reactions and supports the maturation of regenerated tissues. The mechanical properties of PCL, including its flexibility and strength, can be fine-tuned to match the requirements of the nerve tissue. The inherent elasticity of PCL makes it suitable for soft tissue applications, allowing it to conform to the dynamic environment of regenerating nerves. Moreover, PCL can be blended with other materials, such as collagen, chitosan, or PGA, to create hybrid scaffolds with improved mechanical and biological characteristics [87]. These composites combine the advantages of PCL durability with the bioactivity of natural materials, resulting in enhanced outcomes in nerve repair. PCL also serves as an excellent platform for the delivery of bioactive molecules. Growth factors, drugs, or peptides can be encapsulated within PCL scaffolds and released in a controlled manner, creating a bioactive microenvironment that promotes nerve regeneration. For example, neurotrophic factors, such as NGF, can be loaded into PCL-based conduits to enhance axonal outgrowth and neuronal survival. This controlled release capability enables the sustained delivery of therapeutic agents at the injury site and addresses challenges, such as inflammation or insufficient neurotrophic support [88]. In addition to its direct role in nerve repair, PCL may be functionalized to enhance its interaction with cells and tissues. Surface modifications, such as the incorporation of cell-adhesive peptides or plasma treatment, can improve cellular attachment and proliferation on PCL scaffolds [89]. These modifications ensure that the material is biologically active and capable of supporting the cellular processes required for successful nerve regeneration. In addition to having controllable mechanical and degradation characteristics, synthetic polymers PGA and PCL have consequently been used to load drugs, nanoparticles, and stem cell therapies [90,91].

### 6.3. Polylactic Acid (PLA)

PLA is a commonly used synthetic bioactive material for regenerative medicine, particularly for nerve injury and tissue engineering [92,93]. It is a biodegradable, biocompatible thermoplastic made from renewable feedstock, such as corn starch or sugarcane, and has been approved by the FDA for biomedical use [94]. Because of its modularity, which allows it to be manufactured as fibers, thin films, scaffolds, and 3D-printed structures, it is ideal for use in nerve regeneration [95]. Considering the high density of PLA, it affords high mechanical strength and a controlled degradation rate necessary for scaffolding and structural support during tissue repair. The application of PLA for nerve repair takes advantage of its properties as a scaffold to promote axonal regeneration and cell function [96]. Thus, PLA scaffolds can favorably reorganize the surrounding cellular environment by offering structural support and a surface for the attachment, migration, and proliferation of everything from Schwann cells and neurons to other requisite supportive cells. Schwann cells of peripheral nerves involved in nerve regeneration express neurotrophic factors, including NGF and BDNF. There is increased activation of the Schwann cellular profile, proliferation, and induction of axonal growth and myelination by PLA scaffolds [96]. PLA is a compostable material, making it a desirable biodegradable plastic [97]. The hydrolysis of its ester bonds forms lactic acid, which is a natural metabolite in the human body. The degradation rate of PLA can also be controlled by varying its molecular weight or through its copolymerization with other polymers. This tunable degradation profile affords the mechanical properties of PLA scaffolds during the critical phases of endoneurial nerve regeneration and biodegradation during the subsequent tissue remodeling phase [98]. The mechanical requirements for nerve repair can be engineered into PLA through its formulation. Although PLA is rather rigid compared with other synthetic polymers, it is suitable for various applications in which structural performance is required. However, in soft tissue, such as nerves, its brittleness is a limitation because of the inherent rigidity [99]. To overcome this shortcoming, PLA may be copolymerized with other flexible polymers, such as PCL, or combined with natural biomaterials, including collagen or gelatin, because of increased flexibility and bioactivity [99]. Moreover, PLA is an efficient carrier of bioactive molecules. Therefore, growth factors, drugs, or signaling peptides can be incorporated into PLA scaffolds and released on a controlled basis at the injury site. For example, scaffolds containing NGF or VEGF promote axonal regrowth or angiogenesis. The incorporation of these factors creates a more prolonged therapeutic effect and promotes tissue regeneration. Further studies of the PLA surface will improve its performance for nerve repair applications. Techniques, such as plasma treatment, chemical grafting, or the use of bioactive coatings on the scaffold surface, can enhance surface hydrophilicity, cell adhesion, and bioactivity. These changes will enable PLA scaffolds to engage with cells and tissues to improve tissue regeneration [100].

### 6.4. Hybrid Materials

Hybrid materials have emerged as a promising class of biomaterials in nerve regeneration, combining the beneficial properties of both natural and synthetic components to overcome the limitations associated with each material type individually. Natural polymers such as collagen, chitosan, and silk fibroin offer excellent biocompatibility and biological signaling but often lack mechanical strength and structural stability. On the other hand, synthetic polymers like PLGA and PCL provide tunable mechanical and degradation properties but may provoke inflammatory responses or lack cell-specific bioactivity. By integrating these materials into hybrid scaffolds, researchers aim to create nerve guidance conduits that mimic the ECM more closely, enhance Schwann cell adhesion and proliferation, and support axonal outgrowth. Recent studies have shown that hybrid systems, such as collagen–PCL and chitosan–silk fibroin composites, not only improve the biomechanical performance of nerve scaffolds but also reduce immunogenicity and support the controlled delivery of growth factors and stem cells. These innovations position hybrid materials as a critical component in the next generation of personalized and effective nerve repair strategies [101]. They advance regenerative medicine because of their capacity to deliver enhanced biocompatibility, bioactivity, and overall structural strength. Collagen/PCL is a classic hybrid biomaterial used in tissue engineering. As a natural material, it provides the required number of bioactive sites in the scaffold that promote cell adhesion, proliferation, and the alignment of the axonal growth direction [102]. Furthermore, PCL contributes to the mechanical strength required for the long-term stability of the scaffold structure, while providing the biological characteristics of collagen. This hybrid material enhances the threshold and function of Schwann cells, stimulates the secretion of neurotrophic factors, such as NGF, and offers structural stability for the establishment of a regenerative pathway for axons [103]. They progressively degrade and readily incorporate into tissues with minimal inflammation. Another interesting hybrid material is chitosan/PLA combined material. Natural chitosan is biocompatible and bioactive and exhibits a bacteriostatic nature resulting from its cationic nature [104,105]. The scaffolds synthesized by blending PLA, a synthetic polymer with remarkable mechanical strength and a controllable degradation profile, provide improved biological and structural properties. Chitosan promotes the growth of Schwann cells and increases the synthesis of neurotrophic factors. PLA fulfills the role of an organizer necessary for nerve healing [106,107]. PLA degradation products and the bioactivity of chitosan promote axonal regeneration and exclude infections at the lesion site. Another promising hybrid material incorporating silk fibroin and PGA also shows potential in nerve regeneration [108]. The silk fibroin natural protein facilitates cell adhesion and improves the alignment of Schwann cells, while its degradation rate is similar to that of the ECM [109,110]. However, PG degrades faster, thus providing early mechanical reinforcement post-surgery during the regeneration period [111]. This combined scaffold encourages axonal regeneration through the availability of the reduced porosity required for cell invasion, nutrient exchange, and vessel formation. Importantly, the rate of scaffold degradation for silk fibroin and PGA is similar, so the material does not have to be replaced, and instead, it may be displaced by the regenerated tissue in a manner conducive to efficient and long-lasting nerve repair [83]. Overall, hybrid materials provide a natural–synthetic interface to overcome the various challenges in nerve repair. Their bioactivity, mechanical stability, and biodegradation profile provide a suitable environment for cell function and tissue formation [112]. Their capacity to recapitulate the ECM, while promoting long-term tissue remodeling, indicates that they represent promising tools for boosting regenerative medicine or as solutions for nerve injuries.

All major bioactive materials are summarized in Table 1. 

## 7. Smart Tools for Neural Repair: CRISPR and 3D Bioprinting as Catalysts of Change

Smart tools for neural repair represent cutting-edge innovations that integrate biotechnology, nanotechnology, and materials science to support the healing of neurological damage. These technologies are adaptive hydrogels, engineered nanomaterials, and micro/nanofiber scaffolds. These are crafted to interact dynamically with the nervous system, encouraging tissue regeneration and restoring function. By mimicking the natural neural environment and responding to physiological cues, they offer promising avenues for treating injuries and neurodegenerative conditions.

CRISPR tools like CRISPRi and CRISPRa allow the precise control of gene expression, helping to uncover disease mechanisms. A novel approach to peripheral nerve regeneration is using a CRISPR activation (CRISPRa) system delivered via a hybrid baculovirus (BV) vector. The BV-CRISPRa system was designed to simultaneously activate three key neurotrophic genes—BDNF, GDNF, and NGF—in rat adipose-derived stem cells (ASCs). The engineered ASC sheets showed prolonged gene activation and significantly promoted Schwann cell migration, neuron growth, and neurite extension in vitro. When implanted into rat sciatic nerve injury sites, these CRISPRa-activated ASC sheets enhanced nerve repair, including axonal regeneration, remyelination, and functional recovery. This method shows strong potential for gene-activated, stem cell-based nerve regeneration therapies [122]. CRISPR-based functional genomics can be used to understand and treat neurological diseases. High-throughput CRISPR screens can identify causal genetic changes and potential therapeutic targets. This approach holds promise for advancing treatments for neurodegenerative, neurodevelopmental, and neuropsychiatric disorders [123]. Nondividing human neurons repair CRISPR-Cas9-induced DNA damage slowly and differently than dividing cells, using unexpected repair pathways. Researchers found that by adjusting these pathways, gene editing in neurons could become more precise, offering potential for better treatments of neurological diseases. Using iPSCs and iPSC-derived neurons, researchers found that neurons took weeks to repair such damage, unlike iPSCs, which did so in days. Surprisingly, neurons activated DNA repair genes typically linked to DNA replication. By chemically or genetically altering this response, researchers were able to steer repair outcomes toward more precise genome edits. These findings reveal important differences in DNA repair between dividing and nondividing cells. The study provides new insights for improving genome editing in neurons for therapeutic applications [124]. The integration of pharmacogenomics, artificial intelligence (AI), and CRISPR is transforming precision medicine. Pharmacogenomics helps tailor drug therapies based on individual genetic profiles, improving efficacy and reducing side effects. AI enhances biomarker discovery, drug optimization, and predictive modeling. CRISPR enables accurate gene editing to treat various diseases, including cancer and neurological disorders. AI also improves CRISPR precision by minimizing off-target effects. Despite these advances, challenges such as ethical concerns, data privacy, and legal issues must be addressed for broader clinical application [125].

Three-dimensional (3D) printing has seen significant progress, especially in medical applications. A major advancement enabling 3D bioprinting is the use of specialized biomaterials, viable cells, and supportive components to create functional tissues. It involves the layer-by-layer fabrication of biological structures using computer-aided design (CAD) and specialized bioprinters. Unlike conventional 3D printing, which relies on plastics or metals, bioprinting employs bioinks, a mix of living cells, hydrogels, and bioactive molecules to produce tissue-like architectures. Various 3D bioprinting techniques such as laser, inkjet, and extrusion-based methods are used in generating artificial blood vessels and tissue grafts. Progress in additive manufacturing technologies, imaging methods, biomaterial science, and cell-based engineering continues to drive innovation in the creation of personalized vascular tissue models. Continued interdisciplinary efforts are anticipated to further revolutionize tissue engineering and regenerative healthcare solutions [126].

Concerning scalability, recent developments in manufacturing, such as 3D bioprinting, electrospinning, and microfabrication, may facilitate the synthesis of large quantities of bioactive materials with desired properties [127]. After years of research, nerve guides have become a clinical option for short nerve injuries, but challenges remain for long-gap repair. Three-dimensional printing offers promising solutions by enabling customizable nerve guides, internal scaffold fabrication, the bioprinting of cells, and precise growth factor gradients. Advances in printing resolution allow the creation of fascicle-like structures that improve nerve guidance. Ultimately, 3D printing aims to develop patient-specific constructs that outperform traditional autografts in complex nerve regeneration [128]. Nerve repair is challenging due to the limited regenerative capacity of neural tissue. While conventional therapies offer limited success, tissue engineering provides a promising solution through scaffold implantation. The integration of 3D printing allows the precise fabrication of neural structures, enhancing regenerative outcomes [129]. Three-dimensional printing offers innovative solutions for developing neural regeneration devices with anatomical precision and multifunctional capabilities. These devices can incorporate physical, biological, and biochemical cues to support nerve repair without additional surgeries. The technology enables precise spatial control, biomolecule integration, and compatibility with 3D scanning and modeling. Such platforms hold promise for creating personalized, implantable scaffolds and advanced in vitro models. One report reviews recent advancements and future directions in 3D printing for neural repair and regenerative medicine [130]. A biomaterials and regenerative medicine study explored and designed 3D-printed hydrogel which can be used to repair damaged nerve tissues in living organisms. Water-based biodegradable polyurethane dispersions (PU1 and PU2) are used for neural tissue engineering. These hydrogels gel are at body temperature (~37 °C) without toxic crosslinkers, and their stiffness can be adjusted by changing solid content. Neural stem cells (NSCs) embedded in 25–30% PU2 hydrogels showed superior proliferation, differentiation, and neural repair efficacy in zebrafish models compared to those in PU1. Notably, PU2-based constructs successfully restored nervous system function in both embryonic and adult zebrafish with brain injuries. This technique offers promising potential for clinical neural regeneration applications [131]. Traditional drug discovery faces high failure rates in late stages, making the early prediction of drug efficacy and toxicity essential. Advanced in vitro models, especially 3D bioprinted systems, mimic native tissue environments better than 2D models, improving drug screening accuracy. Three-dimensional bioprinting also supports the development of vaccines, gene delivery systems, and regenerative therapies by enabling the precise spatial and chemical replication of tissues. Combining bioprinting with non-viral nanocarriers allows for targeted and controlled gene delivery. Despite these advances, challenges remain in replicating fully functional live tissues in vitro. Overall, 3D bioprinting holds significant promise for enhancing drug discovery, personalized medicine, and therapeutic development [132].

**Figure 3:**
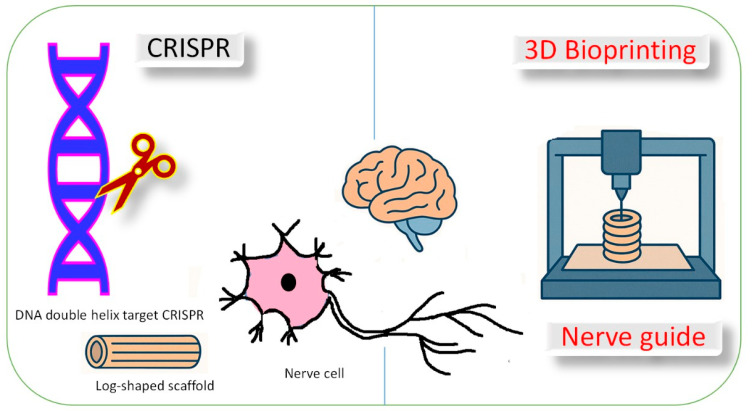
This infographic effectively conveys that CRISPR and 3D bioprinting are complementary technologies driving the next generation of therapies for neural repair and regeneration. CRISPR edits and enhances the biological components, while 3D bioprinting recreates the physical environments needed to support these changes, together enabling precise, patient-specific treatments for neurological injuries and disorders.

CRISPR and 3D bioprinting are complementary technologies with transformative potential in tissue engineering and regenerative medicine. CRISPR, as a precise gene-editing tool, can be employed to genetically modify cells before their integration into 3D bioprinted scaffolds, thereby enhancing their regenerative properties or introducing desired biological functions. The integration of these technologies enables the fabrication of bioengineered tissues with superior therapeutic efficacy and paves the way for advanced applications in disease modeling, drug screening, and personalized treatment strategies. This convergence represents a significant advancement in the field of regenerative medicine and holds great promise for the future of neurotherapeutics [132] (Figure 3).

## 8. Nanotechnology in Nerve Regeneration

The structural and functional complexity of nervous tissue presents significant challenges in developing effective regenerative treatments. While current clinical approaches—such as autografts and stem cell therapies—offer some benefits, options with proven, widespread efficacy remain limited. Recent research has highlighted the promising biological characteristics of nanomaterials, including their excellent biocompatibility, biodegradability, permeability, mechanical strength, and capacity for high drug-loading efficiency [133]. These features make them highly suitable for a wide range of biomedical applications. In the context of peripheral nerve repair, nanomaterials are particularly valuable as they can serve as nerve conduits that provide structural support and directional guidance for regenerating nerves. Additionally, they can be functionalized with therapeutic agents such as cytokines, stem cells, or pharmaceuticals to enhance regenerative outcomes. Most current studies in this area have concentrated on exploring various types of nanomaterials and their specific roles in facilitating peripheral nerve repair [134].

Recent advances in nanotechnology, however, have opened new avenues for personalized therapies targeting both the CNS and PNS. Biocompatible nanomaterials, including nanoparticles, nanogels, membranes, and scaffolds, are being developed as versatile platforms for delivering neuroprotective agents, growth factors, genes, and stem cells. These technologies show strong potential to enhance neuroregeneration by enabling controlled, localized therapeutic delivery [135].

## 9. Advantages of Nerve Repair Technologies

Axonal regeneration and neuronal remodeling are highly intricate and complicated. Therefore, there is a need for a comprehensive method of managing the structural, biological, and functional aspects of the nerves [136]. Bioactive materials have become the basis for creating new technologies for nerve grouping, conduits, hydrogels, nanoparticles, and bioactive coatings. This review discusses these applications, their function, and possibilities for improving nerve regeneration. Nerve conduits and scaffolds are widely used approaches to achieve gap bridging in damaged nerves and to guide the regeneration of axons. Alginate- and collagen-based conduits have bioactivity. They replicate the ECM and promote Schwann cell adhesion, while also encouraging the orientation of axons [137]. Similarly, there are antibacterial conduits of chitosan that implant biocompatibility to form an effective niche for tissue regeneration. Conduit materials include synthetic materials, such as PGA, and PCL conduits, which provide stability and the capability of controlling the degradation period, which is an important issue for long-term support. Some scaffold composites are made from biological and synthetic materials, such as collagen and PCL. Moreover, commercially available decellularized nerve allografts, such as Avance^®^, offer biologically functionalized and prepared scaffolds that maintain the ECM of the nerve fibers to promote functional restoration [138]. These conduits and scaffolds can also be supplemented with electrospun nanofibers, fibrin fillers, or growth factors to enhance their regenerative capability. Hydrogels and injectable biomaterials are used because of their simple administration method and capacity to develop ECM-mimicking matrices. Of these, alginate hydrogels are the most suitable for the immobilization of microorganisms. These hydrated structures enable cell adhesion and the exchange of nutrients. Novel GelMA hydrogels incorporating bioactive peptides enhance Schwann cell proliferation and endothelial cell sprouting [139]. Chitosan-based hydrogels containing neurotrophic factors enhance regeneration [140], whereas self-assembling, peptide-type hydrogels create a stable three-dimensional scaffold that promotes cell invasion [141]. Biodegradable thermo-sensitive polymers, such as Pluronic^®^, gel at physiological temperature, thus allowing the localized controlled release of the active species. The applicability of hydrogels has further expanded by the compatibility of stem cell therapies, including mesenchymal stem cells, which boost the regeneration capabilities of hydrogels [142]. Approaches using nanoparticles and drug delivery systems can provide the high accuracy and stability of released therapeutic agents in nerve repair [143]. Using PLGA opening-coflexible nanoparticles is a frequently used method for the delivery of neurotrophic factors, such as NGF and BDNF, with a sustained release formulation and minimal systemic abscess effects [144]. Chitosan nanoparticles improve drug administration, while exhibiting inherent biological activity and antibacterial properties [145]. Gold nanoparticles conduct electricity and support the growth of neurons, whereas lipid-based nanoparticles can deliver small interfering RNA (siRNA) to modify genes that support regeneration [146]. Nanoparticle drug delivery carriers use cell-derived exosomes to improve intercellular communication to support repair functions. These nanoparticles may be functionally modified to include peptides, drugs, or growth factors as needed [147]. Electrical stimulation and bioactive coatings are two sophisticated applications of nanotechnology developed to improve the cellular compatibility and improve nerve regrowth in damaged regions [148]. Using polypyrrole (PPy), which is a conductive polymer, the electrical signals necessary for promoting neuronal activity and axonal extension in scaffolds are collected. Graphene-based coatings stabilize the properties of neural interfaces by imparting conductivity and biocompatibility to the surface [149]. Laminin or fibronectin bioactive coatings enhance the interfacial cellular response on artificial surfaces. Hydroxyapatite or bioactive glass coatings for neural tissue attachment and structural implants, such as titanium, have also been evaluated [150]. Some of the electrically conductive hydrogels, such as carbon nanotube-infused hydrogels, enhance the effects of electrical stimulation by providing a reactive and conducive environment for growing axons [151].

## 10. Fabrication and Functionalization Process of Nerve Guide Conduits (NGCs)

**Figure 4:**
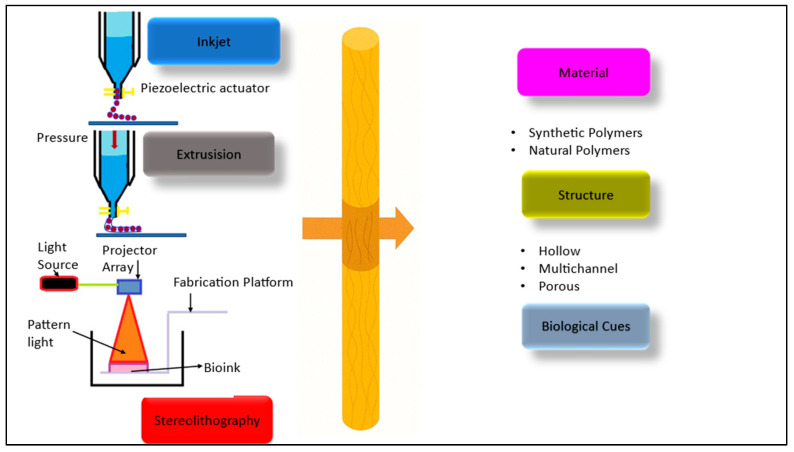
Preparation and functionalization of nerve guide conduits.

Various fabrication methods, including conventional and rapid prototyping techniques, are discussed alongside the influence of features like channel geometry, pore structure, and hydrogel use. While progress has been made, challenges such as axonal misdirection and incorrect reinnervation remain. Unlike the traditional fabrication methods of nerve guidance conduits (NGCs), newer approaches focus on enhancing precision, structural complexity, and biological functionality. Conventional methods often result in limited control over internal architecture and may not fully replicate the native nerve environment. In contrast, advanced techniques like 3D bioprinting and rapid prototyping allow for the creation of customized, biomimetic structures with integrated guidance cues and controlled porosity, improving the potential for effective nerve regeneration [152]. Three-dimensional printing offers the capability to produce NGCs with tailored features that closely replicate the natural architecture of peripheral nerves. Using computer-aided design (CAD) in conjunction with medical imaging techniques such as CT or MRI, the detailed structural information of native tissue can be digitized to guide the fabrication process [153]. Through rapid prototyping, NGCs are constructed layer by layer with high precision. Common 3D printing techniques employed include extrusion-based printing, inkjet printing, and stereolithography, while indirect 3D printing involves creating molds to generate conduits with diverse internal configurations. This approach enhances customization and structural complexity in nerve repair scaffolds [154]. The development of functional NGCs serves as a promising alternative to nerve autografts and allografts for treating peripheral nerve injuries. Although NGCs have shown potential, their efficiency in promoting nerve repair remains limited, necessitating enhancements in their design. Three-dimensional printing offers significant advantages for creating customized, complex, and biomimetic NGCs, improving axonal growth and myelination. The review discusses various 3D printing techniques—inkjet, extrusion, stereolithography, and indirect printing—used in NGC fabrication (Figure 4). It also summarizes key strategies for improving NGC functionality, such as architectural design, material selection, and the incorporation of biological cues [154].

Inkjet printing, also known as drop-on-demand printing, enables the accurate placement of bioinks using thermal or piezoelectric forces. It offers a resolution range of 1–500 μm, influenced by nozzle size and ink properties. Studies show that materials like polylactic acid and poly(ε-caprolactone) can be used to create nerve guidance conduits with high precision. Moreover, neuronal and Schwann cells retain viability when printed, highlighting the technique’s promise for nerve tissue engineering applications [155]. Extrusion bioprinting constructs 3D scaffolds line-by-line using pneumatic or mechanical dispensing systems with a resolution of 100–500 μm. It differs from inkjet printing in that it allows for continuous material deposition. A custom microextrusion system was used to print bifurcated silicone NGCs, improving sciatic nerve repair in rats. Conductive NGCs with fine tips were also printed for repairing nerve defects in human cadavers. Additionally, scaffolds embedded with Schwann cells and HUVECs using gelatin and alginate have been developed. These approaches show strong potential for peripheral nerve regeneration [156]. Bioprinting is transforming healthcare by enabling precise control over cells, genes, proteins, and bioactive compounds to create functional tissues. This technology is seen as a solution to major clinical challenges such as organ transplantation limitations and immune rejection. Hydrogels are emphasized as essential bioinks due to their excellent biocompatibility, tunable physical and chemical properties, and ability to replicate the extracellular matrix (ECM). These features make hydrogels ideal for promoting cell adhesion, growth, and differentiation. The review discusses how hydrogels can be modified through surface functionalization, mechanical tuning, and bioactive molecule incorporation to suit specific tissue engineering needs. Both natural polymers (like collagen, gelatin, fibrin, alginate, hyaluronic acid, chitosan, agarose, and carrageenan) and synthetic polymers (such as PEG, PLA, PLGA, and poloxamers) are described as key components for formulating 3D-printable hydrogels. Choosing the right hydrogel depends on the intended application, whether it be for bone, cartilage, heart, or nerve tissue regeneration. The knowledge of each polymer’s properties, strengths, and limitations is crucial to the success of bioprinting in personalized medicine and functional tissue construction [157]. Nerve guidance conduits for nerve repair can be fabricated with various structural designs, such as hollow, multichannel, or porous forms, and may include biological components like cells, growth factors, or therapeutic agents. These features play a critical role in replicating the native nerve environment, thereby affecting axon regeneration, nutrient transport, and the overall effectiveness of nerve healing. Peripheral nerve injuries often result in motor and sensory dysfunction, and current treatments like nerve autografts have significant limitations. NGCs have emerged as a promising alternative, offering structural and biochemical cues to support axonal growth and nerve repair. Advanced NGCs are now designed to closely mimic the native nerve environment using innovative biomaterials, precise structural designs, and modern fabrication techniques such as 3D printing. These improvements enhance cellular behaviors like adhesion, alignment, and migration, ultimately promoting more effective neural regeneration and functional recovery [112].

## 11. In Vitro and In Vivo Studies

Preclinical and clinical studies are important for enhancing the use of bioactive materials for nerve repair. Moreover, animal studies have provided evidence for the effectiveness of these materials in encouraging the regeneration of nerves and functional recovery [158]. For example, conductive structural collagen conduits have been evaluated in rodents, whereas conduits enhanced axonal regeneration and restored motor function following sciatic nerve injury [159]. Similarly, chitosan scaffolds, which are used to deliver neurotrophic factors, have shown efficacy in rabbit models with respect to nerve repair and reduced inflammation. These studies suggest the use of natural bioactive matrices for the management of nerve injuries because of the ability of the matrices to ensure a suitable environment for healing [160]. Synthetic bioactive materials are also effective in preclinical tests. For example, polyglycolic acid conduits have been studied in primate models for long-gap nerve defects by bridging nerve gaps and restoring sensation [161]. Schwann cell-laden silk fibroin hydrogels exhibited optimal regenerative prospects in mouse models of sciatic nerve injury by hastening regeneration and enhancing coordinated movements [162]. This has been advanced by the use of nanoparticles, such as PLGA-loaded neurotrophic factors, which provide the controlled release of the drug, with more targeting of axonal growth and less neuronal apoptosis [163]. These examples support the capacity of bioactive materials to address the various problems of nerve repair. Focusing on the next steps that connect the preclinical field with clinical applications by translating the results of laboratory studies into practice has accelerated. Nerve allografts, in which cells and cell components are removed, have also provided a clinically applicable nerve graft solution. For example, Avance^®^ nerve allografts improved the sensory and motor function of patients with PNI [138]. Clinical studies of silicone-based nerve conduits have revealed their utility in short distal nerve defects; however, problems, such as nerve conduit stiffness and foreign body response, have limited their use [164]. NeuroGen^®^ is a collagen-based material that has enjoyed reasonable success in digital nerve repair surgeries. It is biocompatible and, most importantly, available for autografts [165]. Hydrogels have also been used clinically to treat spinal cord injuries [166]. Injectable chitosan hydrogels containing mesenchymal stem cells have entered clinical trials and have demonstrated good safety and efficacy in the formation of new neural connections and reducing scar tissue formation [167]. Similarly, other specialized technologies, such as graphene-like electrodes, can restore nerve function by electrical stimulation. Some pilot studies indicate that such coatings improve conductivity and biocompatibility with neural tissue, which suggests future use in neuroprosthetics [168].

In a case study the surgical excision of the neuroma followed by regenerative nerve grafting (RNG) was planned. RNG is an emerging technique offering sustained symptom relief, particularly suited for distal hand neuromas where options like targeted muscle reinnervation (TMR) and regenerative peripheral nerve interfaces (RPNI) may be limited due to insufficient soft tissue. Regenerative nerve grafting provides a scaffold for axonal regeneration, enables the positioning of the nerve end into a supportive tissue bed, and helps disperse axonal signals, reducing the likelihood of neuroma recurrence [169]. Ongoing advancements in surgical tools and techniques have solidified autografting as the gold standard for nerve reconstruction, while also ushering in the era of nerve transfer procedures. Simultaneously, progress in bioengineering has expanded the therapeutic arsenal with the development of implantable devices, including nerve conduits and acellular allografts [170].

Despite progress, the jump from laboratory testing to clinical application is not without problems; however, there are high regulatory barriers because bioactive materials are subject to high safety and efficacy requirements for approval. For example, advances in hydrogel-based drug delivery have slowed because of issues related to stability and possible adverse effects [171]. It is also challenging to scale up the manufacturing of bioactive scaffolds. Uniformity and quality in large-scale fabrication, particularly in structural scaffolds that incorporate natural ECM components and synthetic materials, pose several technical challenges. The heterogeneity of immune responses in humans also must be considered for the clinical application of bioactive materials [172]. Chitosan hydrogels, which have provided positive results in animal models, can cause hypersensitivity reactions in patients [173]. These unpredictable responses call for the additional tailoring of material properties and composition. Furthermore, the high cost of some of the more novel biomaterials, such as PLGA nanoparticles or graphene electrodes, has prompted controversies over their use in clinical applications [174]. The lack of long-term outcome data is another emerging concern that has recently been attributed to clinical trials and scientific research. The initial advantages of conduits, such as silicone conduits, are evident, but long-term studies showed failure in conduit collapse or the formation of scar tissue. Thus, clinical trials with long follow-up periods are needed to evaluate the durability and effectiveness of nerve repair technologies [114]. These limitations will arise as new approaches improving the translational effectiveness of bioactive composites are evaluated. Translating a discovery from the lab to the clinic is not linear but a recursion from the lab, clinic, or regulatory agency. In the future, dissimilarities regarding potential bioactive materials that are indispensable in nerve repair will need to be discussed. Furthermore, challenges must be addressed that impact repaired nerve tissue, such as scalability, cost, the immune response, and long-term efficacy. Further improvements in material design, coupled with extensive in vitro and in vivo studies, will lead to the successful development of novel nerve injury therapies. It is this collective effort that brings hope for significant progress in regenerative medicine that can benefit patients who have suffered severe nerve damage. Table 2 summarizes various in vitro and in vivo studies demonstrating the role of bioactive materials in nerve regeneration, while ongoing clinical trials are presented in Table 3.

Bioactive materials play a crucial role in nerve repair and regeneration by modulating the cellular microenvironment and promoting neural tissue recovery. These materials can enhance cell adhesion, proliferation, and differentiation, particularly those of Schwann cells and neural stem cells, which are vital for axonal guidance and remyelination [186]. Additionally, bioactive scaffolds functionalized with neurotrophic factors or conductive elements have been shown to facilitate synaptic reconnection and improve electrophysiological outcomes in peripheral and central nervous system injuries [187,188].

The integration of such materials into nerve conduits or hydrogels further supports tissue regeneration by providing mechanical support and the controlled release of therapeutic agents. For instance, biomaterials like chitosan, collagen, and polypyrrole have demonstrated promising results in enhancing axonal growth and functional recovery in vivo. These findings underscore the potential of bioactive materials as effective therapeutic platforms for promoting nerve regeneration.

Despite promising advances in preclinical studies, the clinical translation of bioactive materials for nerve regeneration remains limited due to several critical challenges. These include variability in injury models, a lack of standardized evaluation protocols, and differences in anatomical and physiological regeneration capacity between animals and humans [189]. Additionally, many bioactive constructs, such as hydrogels, nanoparticles, or electrospun fibers, face hurdles in regulatory approval due to concerns regarding long-term biocompatibility, scalability, and reproducibility in manufacturing processes [190]. Another major gap lies in the insufficient number of well-powered, randomized clinical trials evaluating these materials in human subjects, which impedes evidence-based validation and wider adoption in clinical practice. Bridging these gaps requires not only robust interdisciplinary collaboration but also the integration of translational frameworks that address regulatory, biological, and engineering perspectives.

## 12. Advantages and Limitations

This review describes how bioactive materials have revolutionized the field of nerve repair. These materials promote tissue repair by providing a structure similar to the ECM that supports scaffold axonal regeneration. This is achieved by improved cell adhesion, proliferation, and differentiation, particularly for Schwann cells, which are required for the myelination and regeneration of nerves. Furthermore, these bioactive materials can be functionalized to deliver growth factors. For example, NGF or BDNF can enhance tissue repair and improve functional recovery. These advantages make bioactive materials essential for nerve repair, particularly under conditions in which autograft surgery cannot provide an adequate solution because of the size of the injury or the location in the body. The blending of bioactive materials with functional electrical stimulation, stem cell therapies, and drug delivery systems has shown great potential when applied to nerve regeneration [186]. However, like any other material that has been proposed for use in clinical settings, bioactive materials have several limitations, such as immunogenicity, which can significantly differ depending on the selected material. For example, collagen and chitosan are natural materials, and although they are biocompatible in most cases, they can cause immune reactions, such as inflammation and hypersensitivity. This may adversely affect nerve repair or may even lead to rejection of material [191]. Bioactive materials have significantly advanced the field of nerve repair by providing structural and biochemical support that mimics the native ECM, thereby enhancing axonal regeneration. These materials support Schwann cell adhesion, proliferation, and differentiation—key steps in myelination and nerve regeneration. Moreover, their ability to be functionalized with neurotrophic factors such as NGF or BDNF further enhances regenerative outcomes and accelerates functional recovery. Bioactive materials are particularly advantageous in clinical scenarios where autologous nerve grafts are not feasible due to injury length, location, or donor site limitations. Additionally, their integration with technologies such as electrical stimulation, stem cell therapy, and controlled drug delivery has further expanded their therapeutic potential [192].

Despite these advantages, several limitations remain. One of the foremost challenges is immunogenicity. While natural polymers such as collagen and chitosan are widely used due to their biocompatibility, they can still trigger immune responses, particularly when sourced from xenogeneic materials. For instance, collagen scaffolds derived from bovine or porcine sources may carry residual antigens that can provoke macrophage infiltration and chronic inflammation, ultimately hindering nerve repair [193]. Similarly, chitosan, although derived from natural polysaccharides, has been associated with hypersensitivity reactions in some cases [194]. In contrast, synthetic materials such as PLGA or PCL tend to be less immunogenic, but their degradation products—such as lactic and glycolic acids—can accumulate locally, leading to acidic microenvironments that promote inflammation and impair tissue regeneration [81].

To mitigate immunogenic responses, surface modification strategies have been explored. Coating scaffolds with bioactive peptides (e.g., RGD), proteins, or anti-inflammatory agents can improve cell–material interactions while reducing host immune activation. Additionally, the use of hybrid scaffolds, such as collagen–PCL or chitosan–silk fibroin composites, has shown promise in balancing biocompatibility and mechanical properties while reducing adverse reactions [195]. Another persistent challenge lies in the degradation kinetics of biomaterials. Materials that degrade too rapidly may fail to provide long-term structural support, while those that degrade too slowly may lead to fibrosis or material encapsulation. For example, PLGA-based conduits have shown reduced axonal penetration in vivo due to fibrotic tissue formation surrounding the degrading polymer [196]. Addressing this issue requires the design of materials with tunable degradation profiles. Blending fast- and slow-degrading polymers, using enzyme-responsive crosslinkers, or incorporating biodegradable nanoparticles within scaffolds are all strategies currently being investigated.

Another significant barrier to clinical translation is the challenge of reproducible and scalable manufacturing. While techniques like electrospinning and freeze-drying enable the fabrication of highly porous and bioactive scaffolds at the laboratory scale, scaling up these processes often introduces variability in fiber diameter, porosity, and mechanical integrity. Studies have demonstrated that even minor fluctuations in ambient humidity during electrospinning can lead to inconsistencies in scaffold architecture, affecting cellular behavior and drug release profiles. To address this, automated and closed-loop manufacturing systems, including 3D bioprinting and robotic-assisted scaffold fabrication, are being developed to standardize production while reducing human error and cost [197]. The cost of bioactive materials, especially those involving complex synthesis such as graphene-based nanostructures or functional nanoparticles, remains another limitation. These high-performance materials demonstrate excellent conductivity and bioactivity, but their high production cost limits widespread adoption, especially in resource-constrained healthcare systems. Consequently, researchers are exploring more affordable alternatives such as PCL and PLA—both FDA-approved biodegradable polymers—which offer sufficient mechanical and biological properties at a fraction of the cost. These polymers can be further functionalized to enhance their bioactivity without significantly increasing the expense [93]. Moreover, naturally derived materials like silk fibroin or chitosan are being optimized for use in nerve conduits due to their relatively low cost, ease of processing, and favorable biological performance. Regulatory hurdles represent an additional barrier to clinical implementation. Novel materials often require extensive preclinical testing to evaluate long-term safety, biocompatibility, and efficacy. This regulatory scrutiny, while essential, prolongs the time-to-market and increases development costs. For instance, promising silk fibroin–based nerve guides functionalized with NGF have faced delays in clinical trials due to regulatory requirements for chronic toxicity and immunogenicity studies. Engaging regulatory bodies early in the material development process, using clinically approved base materials, and adhering to GMP standards during manufacturing are essential strategies for overcoming these barriers. Ultimately, the successful clinical translation of bioactive materials for nerve repair will depend on interdisciplinary collaboration between material scientists, biologists, engineers, and clinicians. Emerging technologies such as AI-driven scaffold design, real-time manufacturing monitoring, and bioresponsive material platforms are expected to accelerate progress in this field. At the same time, continued efforts to balance performance, safety, scalability, and affordability will be critical for ensuring the accessibility and sustainability of advanced nerve repair therapies.

These methods enable direct control over the structure/property material relations of the scaffolds, which can be tailored for various nerve repair applications. The use of robotics for the production of bioactive materials and the optimization of the synthesis processes will reduce the manufacturing cost, thus making them more affordable. To overcome the cost issues, researchers are looking into cheap alternatives for materials that provide the required properties of a nerve bridge. For example, PCL or PLA, which are biodegradable polymers, are being evaluated for use as nerve conduits because they are cheap and easy to fabricate compared with other synthetic materials [198]. Furthermore, the application of biodegradable polymers or natural materials, including silk fibroin or chitosan, can be a cost-effective solution without adversely affecting their biocompatibility or tissue repairing ability [199]. Moreover, interdisciplinary strategies, specifically communication between material scientists, biologists, and clinicians, are necessary to create bioactive materials that have long-lasting efficacy at a reasonable price. Such progress is important for maintaining the future of nerve repair therapies and the expenses associated with developing and administering bioactive materials.

Among the various natural and synthetic biomaterials explored for tissue engineering, each offers unique advantages. However, when prioritizing biological outcomes, including cell compatibility, tissue integration, immune modulation, and biofunctionality, the following insights emerge (Table 4).

Hybrid systems, particularly collagen–PCL and chitosan–silk fibroin composites, currently demonstrate the best overall biological outcomes due to their ability to combine biocompatibility, mechanical strength, and biofunctionality. Additionally, electrospun PCL/PLGA fibers and hydrogels offer ECM-mimetic structures and delivery capabilities, further enhancing regenerative outcomes. For next-generation scaffolds, multifunctional composites show great promise but require further in vivo validation.

Neuroprosthetic devices designed for nerve interfaces require precise and stable contact between the electrode and nerve tissue to accurately detect the low-amplitude electrical signals transmitted by axons or to stimulate specific axonal populations. As microfabrication techniques advance, enabling the development of increasingly miniaturized implants, establishing a reliable and long-lasting interface between the implant and neural tissue becomes a critical challenge. Historically, the field relied on rigid materials compatible with standard microfabrication processes. However, there has been a significant shift toward flexible and soft devices that better conform to the dynamic biological environment. While cuff electrodes have been widely used in clinical applications for nerve stimulation, newer technologies—such as the Transverse Intrafascicular Multichannel Electrode (TIME)—have demonstrated promising results in human studies due to their ultraflexible nature. Despite these advantages, the transition to soft neuroprosthetic materials presents considerable fabrication hurdles. Techniques developed for the semiconductor industry, which are optimized for rigid substrates, are not easily adaptable to flexible or soft materials. This issue becomes even more pronounced as implant designs evolve to target smaller neural structures with increased complexity. To address this, researchers are exploring innovative approaches, including the use of thin yet stiff materials that offer the necessary flexibility without compromising structural integrity [200].

## 13. Conclusions

The future of nerve repair lies in integrating cutting-edge technologies with personalized therapeutic strategies to fully harness the potential of bioactive materials. Innovations such as 3D printing and CRISPR-based gene editing hold promise for creating patient-specific scaffolds, cortical cells, and regenerative matrices that enhance functional recovery. Molecular medicine will further revolutionize nerve repair by enabling targeted treatments for genetic and physiological conditions. Additionally, the convergence of bioactive materials with neural interfaces—such as brain–computer interfaces and neuroprosthetics—will help restore neural function more completely. Realizing these advancements will require close collaboration across materials science, molecular biology, clinical practice, and engineering. Through such interdisciplinary efforts, future therapies will be more effective, personalized, and clinically viable, marking a significant leap forward in regenerative medicine for nerve injuries.

## Figures and Tables

**Table 1:** Summary of bioactive materials: properties, characteristics, and applications in nerve repair and regeneration.

Bioactive Material	Properties/Characteristics	Applications in Nerve Repair/Regeneration	Reference
Collagen	Biocompatible, biodegradable, supports cell adhesion and proliferation, mimics ECM	Used in nerve conduits and scaffolds to support axonal growth and Schwann cell proliferation	[52]
Chitosan	Biocompatible, antibacterial, supports Schwann cell adhesion and axonal alignment	Fabricated into scaffolds and hydrogels for peripheral nerve regeneration	[113]
Silk Fibroin	High tensile strength, biocompatible, tunable degeneration, supports nerve cell interaction	Applied in nerve guidance conduits and coatings for scaffolds	[70]
Polylycolic Acid (PLA)	Biodegradable, mechanically strong, readily fabricated into fibers or meshes	Used in synthetic nerve conduits to guide axonal regeneration	[114]
Polycaprolactone (PCL)	Biodegradable, low degradation rate, high mechanical strength	Incorporated into scaffolds and electrospun fibers for long-term nerve repair	[115]
Polylactic-co-glycolic Acid (PLGA)	Biodegradable, adjustable degradation rates, supports drug delivery	Used in controlled release systems for growth factors in nerve repair	[81]
Hybrid Collagen/PCL Scaffold	Combines collagen biocompatibility with the mechanical strength of PCL	Provides structural integrity and a biomimetic environment for axonal growth	[116]
Chitosan–Silk Fibroin Composite	Enhances biocompatibility, improves tensile strength, supports cell proliferation	Used in nerve conduit fabrication for enhanced regenerative potential	[117]
Graphene-Based Materials	Conductive, promotes neural differentiation, has antibacterial properties	Integrated with scaffolds for electrical stimulation and nerve repair	[118]
Hydrogel	Highly hydrated, mimics ECM, supports cell encapsulation	Injectable hydrogels for delivering cells and growth factors to the injury sites	[119]
Nanoparticles	Controlled release of therapeutic agents and high surface area	Used for targeted drug delivery and growth factor release in nerve injuries	[120]
Electrospun Fibers	Mimics ECM structure, customizable alignment diameter	Guides axonal growth and supports Schwann cell migration	[121]

**Table 2:** A summary of in vitro/vivo studies of bioactive materials in experimental models.

Bioactive Material	Study Type (In Vitro/In Vivo)	Experimental Model/Design	Key Findings	Reference
Collagen	In Vivo	Sciatic nerve injury model in rats using collagen scaffolds.	Collagen scaffolds promoted axonal regeneration, limited fibrotic scar formation, and promoted motor and sensory functional recovery of the nerves.	[175]
Chitosan	In Vitro and In Vivo	Rat Schwann cells cultured on chitosan conduits; implantation in sciatic nerve defect models.	Supported Schwann cell attachment as well as animal studies regarding the beneficial effects above; MG niejs indicated that axonal regeneration and the improvement of myelination occurred concomitantly with the minimum stimulation of the immune cells.	[176]
Silk Fibroin	In Vivo	Silk fibroin conduits tested in a 10 mm rat sciatic nerve defect model.	Promoted nerve fiber sprouting and functional regain; the decline rate corresponded to the rate of nerve regeneration, thus reducing the side effects.	[177]
Polyglycolic Acid	In Vivo	A rabbit ulnar nerve defect was repaired using PGA conduits combined with NGF.	Superior morphological and functional outcomes; NGF-conjugated conduits provided superior outcomes compared with conduits only.	[178]
Polycaprolactone	In Vitro and In Vivo	Electrospun PCL fibers aligned to guide axonal growth in dorsal root ganglion (DRG) cultures; implantation in rat sciatic nerve injuries.	PCL fibers aligned in a specific direction facilitated the adherence of Schwann cells and guaranteed directed axonal elongation in vitro and improved functional nerve regeneration in vivo and demonstrated excellent biocompatibility and mechanical stability.	[179]
PLGA	In Vivo	PLGA microspheres loaded with NGF implanted in a sciatic nerve crush injury model in rats.	The chronic release of NGF from PLGA microspheres enhanced neuronal survival and axon elongation and functional restoration over the long-term.	[179]
Collagen–PCL Hybrid Scaffold	In Vivo	Hybrid scaffold tested in a rat sciatic nerve defect model over 12 weeks.	When given together, the mechanical strength and bioactivity were respectable with axonal regeneration, Schwann cell migration, and improved electrophysiological recovery.	[180]
Chitosan–Silk Fibroin Composite	In Vitro and In Vivo	Schwann cells cultured on chitosan–silk fibroin scaffolds; 15 mm sciatic nerve defects in rats repaired with the composite scaffold.	Improved Schwann cell attachment and proliferation in a rat fibroblast growth factor–culture system in vitro; improved axonal regeneration and functional recovery in the transected rat spinal cord in vivo compared to those achieved by single-material scaffolds.	[181]
Graphene-Based Materials	In Vitro and In Vivo	Neural stem cells cultured on graphene-coated surfaces; graphene scaffolds implanted in peripheral nerve injury models.	The networks had better profiles for neural differentiation and supported electrical stimulation for nerve regeneration; graphene supported neurogenesis and was biocompatible.	[182]
Hydrogels (Various)	In Vivo	Injectable hydrogel loaded with mesenchymal stem cells and VEGF tested in sciatic nerve defect models in rodents.	Created a three-dimensional culture for the cells; stimulated blood vessel formation and axon sprouting, thereby enhancing functional rehabilitation.	[183]
Nanoparticles (Various)	In Vitro and In Vivo	PLGA nanoparticles delivering NGF and curcumin to injured sciatic nerves in rats.	Improved the delivery of bioactive molecules to the injury site; significantly enhanced nerve regeneration and functional outcomes compared with direct injections.	[184]
Electrospun Fibers (PCL/PLGA)	In Vitro and In Vivo	Electrospun fibers seeded with Schwann cells were tested in DRG cultures and rat sciatic nerve injury models.	Directed axonal growth in vitro and supported nerve repair in vivo, demonstrating the importance of fiber alignment and material composition for regeneration.	[185]

**Table 3:** Ongoing clinical trials on nerve regeneration using bioactive materials.

Application	Trial	Description	Phase	Status	Trial Number
Use of Nerve Conduits or Scaffolds with Bioactive Components	Nerve Repair Using a Chitosan-based Nerve Conduit	Evaluating a biodegradable chitosan conduit enriched with growth factors for peripheral nerve regeneration after injury.	II	Recruiting	NCT04223545
	Peripheral Nerve Repair Using Collagen-Based Scaffold with Neurotrophic Factors	Investigating the efficacy of collagen scaffolds embedded with NGF (nerve growth factor) to enhance nerve regeneration in traumatic nerve injuries.	II	Active, not recruiting	NCT03793630
Stem Cell-Seeded Bioactive Materials for Nerve Repair	Autologous Stem Cells on a Biodegradable Scaffold for Peripheral Nerve Regeneration	Using autologous mesenchymal stem cells seeded on a bioactive polymer scaffold for the treatment of peripheral nerve defects.	I/II	Recruiting	NCT04652736
	Combined Use of Adipose-Derived Stem Cells and Nerve Guidance Conduits	Examining the safety and preliminary efficacy of ADSCs combined with a synthetic nerve conduit in patients with nerve gaps.	I	Recruiting	NCT05132567
Hydrogel-Based Bioactive Materials for Nerve Regeneration	Injectable Hydrogel Loaded with Neurotrophic Factors for Nerve Regeneration	Testing an injectable hydrogel containing BDNF (brain-derived neurotrophic factor) for the treatment of peripheral nerve injuries	I	Recruiting	NCT05487622

**Table 4:** Summary of bioactive materials.

Bioactive Material	Pros	Cons	Use
Collagen	Excellent biocompatibility and native ECM mimicry; supports cell adhesion and proliferation	Weak mechanical strength and fast degradation	Ideal for soft tissue applications but often combined with synthetic polymers for mechanical reinforcement
Chitosan	Antibacterial, hemostatic, and biocompatible; promotes wound healing	Limited mechanical strength and cell adhesion unless modified	Frequently used in wound dressings, nerve, and cartilage regeneration
Silk Fibroin	Strong mechanical properties, slow degradation, good biocompatibility	Requires purification and can elicit immune response if not processed correctly	Supports bone and ligament regeneration; useful in load-bearing applications
PCA	Fast degradation, suitable for temporary scaffolds	Acidic degradation byproducts can cause inflammation; limited bioactivity	Often used in sutures and rapidly regenerating tissues
PCL	Slow degradation, good mechanical strength, tunable via electrospinning	Hydrophobic and poor cell adhesion unless surface modified	Applied in hard tissue engineering and slow-regenerating tissues
PLGA	Biodegradable, tunable degradation rate, FDA-approved	Can lead to acidic environment; hydrophobic surface	Widely used in drug delivery and scaffold-based regeneration
Collagen–PCL Hybrid Scaffold	Combines ECM mimicry with mechanical robustness; promotes better cell response than PCL alone	-	Particularly effective in bone and skin regeneration due to synergy of natural and synthetic components
Chitosan–Silk Fibroin Composite	Combines bioactivity of chitosan with mechanical strength of silk; improved cell adhesion and proliferation	-	Suitable for neural and skin tissue engineering
Graphene-Based Materials	Antibacterial, conductive (beneficial in neural and cardiac tissue); supports stem cell differentiation	Concerns over long-term safety and in vivo toxicity; still under preclinical exploration	Promising in neural, bone, and cardiac regeneration
Hydrogels	Excellent for mimicking ECM; highly biocompatible; tunable for growth factor delivery and cell encapsulation	Weak mechanical strength unless reinforced	Tissue engineering and regeneration
Electrospun Fibers (PCL/PLGA)	Mimic ECM structure; allow controlled release of bioactive agents	-	Widely used in skin and vascular grafts

## Data Availability

No new data were created or analyzed in this study. Data sharing is not applicable to this article.
